# Association between sex hormone-binding globulin (SHBG) and metabolic syndrome among men

**DOI:** 10.1590/1516-3180.2014.1322666

**Published:** 2014-04-01

**Authors:** Emmanuela Quental Callou de Sá, Francisco Carleial Feijó de Sá, Kelly Cristina Oliveira, Fausto Feres, Ieda Therezinha Nascimento Verreschi

**Affiliations:** I MD, PhD. Endocrinologist, Universidade Federal do Ceará (UFC), Barbalha, Ceará, Brazil; II MD. Cardiologist and Hemodynamicist, Universidade Federal do Ceará (UFC), Barbalha, Ceará, Brazil; III MSc. Pharmacist, Universidade Federal de São Paulo (Unifesp), São Paulo, Brazil; IV MD, PhD. Cardiologist and Hemodynamicist, Instituto Dante Pazzanese de Cardiologia, São Paulo, Brazil; V MD, PhD. Endocrinologist, Universidade Federal de São Paulo, (Unifesp), São Paulo, Brazil

**Keywords:** Sex hormone-binding globulin, Metabolic syndrome X, Men, Coronary artery disease, Coronary angiography, Globulina de ligação a hormônio sexual, Síndrome X metabólica, Homens, Doença da artéria coronariana, Angiografia coronária

## Abstract

**CONTEXT AND OBJECTIVE:**

Metabolic syndrome consists of a set of factors that imply increased risk of cardiovascular diseases. The objective here was to evaluate the association between sex hormone-binding globulin (SHBG), sex hormones and metabolic syndrome among men.

**DESIGN AND SETTING:**

Retrospective analysis on data from the study "Endogenous oestradiol but not testosterone is related to coronary artery disease in men", conducted in a hospital in São Paulo.

**METHODS:**

Men (aged 40-70) who underwent coronary angiography were selected. The age, weight, height, waist circumference, body mass index and prevalence of dyslipidemia, hypertension and diabetes of each patient were registered. Metabolic syndrome was defined in accordance with the criteria of the Third Report of the National Cholesterol Education Program Expert Panel on Detection, Evaluation and Treatment of High Blood Cholesterol in Adults (NCEP-ATPIII). Serum samples were collected to assess the levels of glucose, total cholesterol, HDL-cholesterol (high density lipoprotein), triglycerides, albumin, SHBG, estradiol and total testosterone (TT). The levels of LDL-cholesterol (low density lipoprotein) were calculated using Friedewald's formula and free testosterone (FT) and bioavailable testosterone (BT) using Vermeulen's formula.

**RESULTS:**

141 patients were enrolled in the study. The prevalence of metabolic syndrome was significantly higher in the first SHBG tercile than in the second and third terciles. A statistically significant positive association between the SHBG and TT values was observed, but no such association was seen between SHBG, BT and FT.

**CONCLUSION:**

Low serum levels of SHBG are associated with higher prevalence of metabolic syndrome among male patients, but further studies are required to confirm this association.

## Abstract (pt)

**CONTEXTO E OBJETIVO:**

A síndrome metabólica (SM) consiste em um conjunto de fatores que implicam risco elevado para doenças cardiovasculares. O objetivo foi avaliar a associação entre a globulina ligadora de esteroides sexuais (SHBG), hormônios sexuais e a SM em homens.

**TIPO DE ESTUDO E LOCAL:**

Análise retrospectiva de dados do estudo "Estradiol mas não testosterona se correlaciona com doença arterial coronariana em homens", conduzido em um hospital em São Paulo.

**MÉTODOS:**

Foram selecionados pacientes do sexo masculino com idade entre 40 e 70 anos, submetidos a angiografia coronária. A idade, a prevalência de dislipidemia, hipertensão e diabetes, o peso, a altura, cintura e o índice de massa corpórea de cada paciente foram coletados. A definição de SM seguiu os critérios do NCEP-ATPIII. Amostras séricas foram coletadas para análises da glicose, colesterol total, colesterol-HDL (*high density lipoprotein*), triglicerídeos, albumina, SHBG, estradiol e testosterona total (TT). O colesterol-LDL (*low density lipoprotein*) foi calculado pela fórmula de Friedewald e as testosteronas livre (TL) e biodisponível (TB) pela fórmula de Vermeulen.

**RESULTADOS:**

Entraram no estudo 141 pacientes. A prevalência de SM foi significativamente maior no primeiro tercil de SHBG em comparação ao segundo e terceiro tercis. Foi verificada uma associação positiva e significativa ente os valores de SHBG e TT, porém essa associação não foi verificada entre SHBG e TB e TL.

**CONCLUSÃO:**

Baixos níveis séricos de SHBG estiveram associados com alta prevalência da SM em pacientes do sexo masculino. Faz-se necessário que estudos avaliem essa associação.

## INTRODUCTION

Metabolic syndrome consists of a set of factors that confer increased risk of cardiovascular diseases, including obesity (especially abdominal obesity), insulin resistance (regardless of the presence of diabetes mellitus), dyslipidemia (increased triglyceride levels and reduced HDL cholesterol levels) and systemic arterial hypertension (SAH). The primary abnormality relating to metabolic syndrome appears to be insulin resistance in peripheral tissues.^1^

Sex hormone-binding protein (SHBG), which is produced by the liver, binds to testosterone with high affinity and to estradiol (E_2_) with lower affinity. Insulin is an important regulator of SHBG production in the liver. *In vitro* studies have shown that physiological concentrations of insulin inhibit SHBG production in cultured hepatoma cells.^2^ Pasquali et al.^3^ showed that inhibition of insulin secretion by means of diazoxide induces an increase in SHBG levels, both in obese men and in men with normal body weights. In addition, men who present low SHBG concentrations are at increased risk of developing metabolic syndrome.^4^ The prevalence of concomitant dyslipidemia, hypertension and diabetes suggests that insulin resistance may be a determinant of SHBG levels. 

## OBJECTIVE

This study assessed the association between SHBG, sex hormones and prevalence of metabolic syndrome.

## METHODS

**Study design**

This study was conducted by performing a retrospective analysis on the data from a previous study, "Endogenous oestradiol, but not testosterone, is related to coronary artery disease in men".^5^

## Study population

We selected male patients aged 40-70 years who were admitted to hospital in order to undergo coronary angiography for investigation and/or staging of ischemic heart disease, at Hospital Dante Pazzanese de Cardiologia. Patients who were smokers, were using anti-androgenic drugs (such as ketoconazole, cimetidine, spironolactone, androcur or finasteride) or had a previous history of myocardial infarction, stroke and/or major surgery within the past six months were excluded from the study. In addition, individuals with a body mass index (BMI, defined as weight in kg divided by the square of height in meters) ≥ 40 kg/m^2^ or serum creatinine levels > 2.0 mg/dl, and patients who showed evidence of major liver disease during clinical examinations were also excluded from the study.

After verifying the study inclusion criteria, the patients were divided into three groups based on SHBG terciles.

Informed consent was obtained from all subjects, and the project had previously been approved by the Ethics Committees of the two participating institutions.

## Assessments

Data on age, prevalence of components of metabolic syndrome (obesity, dyslipidemia, SAH and diabetes), current medications, weight, height and waist circumference measurements and BMI were gathered in relation to each patient through standard questionnaires immediately after the individual had been given explanations about the study and had consented to participation in it. Metabolic syndrome was defined in accordance with the criteria recommended by the Third Report of the National Cholesterol Education Program Expert Panel on Detection, Evaluation and Treatment of High Blood Cholesterol in Adults (NCEP-ATPIII). These criteria state that three or more of the following criteria should be present: abdominal obesity (waist circumference > 102 cm in men and > 88 cm in women); triglycerides ≥ 150 mg/dl; HDL cholesterol < 40 mg/dl in men and < 50 mg/dl in women; blood pressure ≥ 130/85 mmHg; and fasting glucose ≥ 110 mg/dl.^6^

## Laboratory analysis

Serum samples were collected between 8:00 AM and 10:00 AM, after overnight fasting, before the coronary angiography. Glucose, total cholesterol, HDL cholesterol, triglyceride and albumin levels were measured in the laboratory of Hospital Dante Pazzanese de Cardiologia. LDL cholesterol was calculated using Friedewald's formula.^7^

Blood samples for hormone determinations were taken to the Steroid Laboratory of Escola Paulista de Medicina, Universidade Federal São Paulo (EPM-Unifesp), immediately after collection. The samples were centrifuged and frozen at -21 °C for no longer than six months. All determinations were performed in duplicate. SHBG was measured by means of the immunofluorometric assay (IFMA; Delfia Perkin Elmer, São Paulo, Brazil), with a detection limit of 0.5 nmol/l. The intra-assay coefficient of variation (CV) was 3.9, 4.9 and 3.3% and the inter-assay CV was 2.3, 3.0 and 2.4%, for 25.5, 63.9 and 138.0 nmol/l, respectively. Testosterone was measured by means of radioimmunoassay with local historical controls, with a detection limit of 0.35 nmol/l, intra-assay CV of 7.5 and 13.2% for 16.7 and 3.86 nmol/l, respectively, and inter-assay CV of 15.5 and 17.6% for 22.87 and 4.97 nmol/l, respectively. The anti-serum used was the anti-testosterone 3-(O-carboxymethyl)-oxime-BSA, and the radioactive standard was (1,2,6,73H)-testosterone (250 µCi) (Amersham Biosciences, Uppsala, Sweden). E_2 _was measured by means of IFMA (Delfia Perkin Elmer, São Paulo, Brazil), with a detection limit of 0.05 nmol/l, intra-assay CV 6.9% and inter-assay CV 9.7%. Free and bioavailable testosterone were evaluated from serum total testosterone (TT), SHBG and serum albumin in accordance with the formula of Vermeulen et al.^8^

## Statistical analysis

Analysis of variance (ANOVA) was used to determine the statistical significance of numerical variables, and Fisher's exact test was used for categorical variables. Data were analyzed using GraphPad Prism 5.0 (San Diego, CA, USA). Relative risk (RR) evaluations were used to assess the prevalence of metabolic syndrome according to SHBG tercile groups. Statistical significance was set at P < 0.05.

## RESULTS

A total of 141 patients were eligible for the study. Increased SHBG levels were negatively associated with BMI, abdominal circumference and prevalence of diabetes (Table 1). No inverse associations between SHBG levels and fasting serum triglyceride levels (P = 0.06) or between SHBG levels and SAH prevalence (P = 0.08) were observed (Table 1). The prevalence of metabolic syndrome was significantly higher in the first (lowest) SHBG tercile than in the second (middle) and third (highest) SHBG terciles (Table 2). A significant positive association between SHBG and TT levels was observed, but no significant associations were observed between SHBG and bioavailable testosterone (BT) or between SHBG and free testosterone (FT) (Table 1). Similarly, no statistically significant association was observed between SHBG and E2 levels (Table 1).

**Table 1.** Laboratory and clinical characteristics of patients according to their sex hormone-binding globulin (SHBG) tercile

	SHBG tercile			P
	1 (lowest)	2	3 (highest)	
SHBG mean (nmol/l) SHBG variation (nmol/l)	27.20 12.2-35.5	43.11 36.1-49.9	72.01 50.5-159	< 0.001
Age (years)*	57.0	56.73	58.88	0.33
BMI (kg/m^2^)*	28.76	27.46	25.13	< 0.001
Abdominal circumference (cm)*	103.49	97.66	93.53	< 0.001
Diabetes (%)	38.30	21.28	8.51	0.002
Dyslipidemia (%)	70.21	63.83	57.45	0.44
Hypertension (%)	95.74	80.85	87.23	0.08
Blood glucose (mg/dl)*	111.04	107.49	99.07	0.42
Total cholesterol (mg/dl)*	183.72	180.30	168.38	0.22
LDL-cholesterol (mg/dl)*	107.24	105.34	98.70	0.51
HDL-cholesterol (mg/dl)*	40.30	41.98	43.34	0.35
Triglycerides (mg/dl)*	174.38	171.28	131.79	0.06
Total testosterone (nmol/l)*	13.63	16.48	20.84	< 0.001
Bioavailable testosterone (nmol/l)*	7.45	7.23	6.61	0.55
Free testosterone (nmol/l)*	0.32	0.31	0.28	0.50
Estradiol (pmol/l)*	68.72	79.14	74.25	0.29

BMI = body mass index

LDL = low-density lipoprotein

HDL = high density lipoprotein

* Data are expressed as means

**Table 2.** Prevalence of metabolic syndrome according to sex hormonebinding globulin (SHBG) tercile

SHBG terciles	1 (lowest)	2	3 (highest)
Metabolic syndrome %	70.21	44.68	19.15
Relative risk	1	0.60	0.29
Confidence interval (95%)		0.06-0.47	0.16-0.54

## DISCUSSION

In this sample of 141 male patients admitted to undergo coronary angiography in order to diagnose coronary artery disease, at Hospital Dante Pazzanese de Cardiologia, SHBG levels presented significant inverse associations with BMI, abdominal circumference and prevalence of type 2 diabetes (Table 1). However, no inverse associations between SHBG levels and fasting serum triglyceride levels (P = 0.06) or between SHBG levels and SAH prevalence (P = 0.08) were observed (Table 1). Furthermore, no significant association between the serum levels of SHBG and HDL cholesterol was observed (Table 1). A significant negative association between SHBG levels and the prevalence of metabolic syndrome was found for patients in the second and third SHBG terciles, with a RR of metabolic syndrome of 0.60 for the second tercile (95% CI: 0.06 - 0.47) and 0.29 for the third tercile (95% CI: 0.16 - 0.54), in comparison with the first tercile (Table 2).

There is increasing evidence in the literature to suggest that low SHBG levels are correlated with components of metabolic syndrome. In a recently published paper, Ding et al.^9^ concluded that low SHBG levels are a strong risk predictor for type 2 diabetes. Similarly, in a meta-analysis, Ding et al.^10^ found that white males with SHBG levels > 28.3 nmol/l presented a 52% lower risk of having diabetes, compared with men with SHBG levels ≤ 28.3 nmol/l. Colangelo et al.^11^ observed a significant inverse association between SHBG levels and abnormal fasting glucose levels and type 2 diabetes among 3,156 men of various ethnicities. Muller et al.^12^ also reported negative correlations between SHBG levels and risk factors for metabolic syndrome. A cross-sectional study by Gannagé-Yared,^13^ which included 201 young males aged 18-30 years, identified significant negative correlations between serum SHBG levels and the levels of triglycerides, HOMA-IR and C-reactive protein (CRP). Finally, in cross-sectional and longitudinal studies on the second and third-generation populations of the Framingham Heart Study, Bhasin et al.^14^ observed that SHBG is an independent predictor for incidence of metabolic syndrome. The cross-sectional evaluations in that study revealed that TT and FT were associated with the prevalence of metabolic syndrome, but a stronger association was observed for TT than for FT. Nevertheless, neither TT nor FT was associated with the prevalence of metabolic syndrome in the longitudinal evaluations. Consequently, these authors concluded that their data did not corroborate the hypothesis that low TT levels were independently associated with the prevalence of metabolic syndrome. 

The primary abnormality observed in metabolic syndrome appears to be insulin resistance in peripheral tissues. Because insulin is a potent inhibitor of SHBG production in the liver, it is possible that decreased levels of SHBG could be an early marker for metabolic syndrome. Similarly, both Heald et al.,^15^ in a study examining European, Pakistani and Afro-Caribbean populations, and Chubb et al.,^16^ in a population-based study, suggested that SHBG is a potential marker for metabolic syndrome. In a recent non-interventional study examining 80 patients with metabolic syndrome, it was reported that an increase of one unit in insulin levels resulted in a decrease of 0.25 units in SHBG levels.^17^

Treatment with the PPARÎ³ agonist rosiglitazone has been shown to increase the blood levels of SHBG, especially in patients with polycystic ovarian syndrome.^18^

Insulin resistance can be defined as a subnormal state of biological responses to circulating insulin. Recent evidence has shown that inflammatory serum mediators may induce insulin resistance.^19^ CRP is the best studied and best characterized inflammatory marker. Kupelian et al.,^20^ in a recent non-interventional population-based study examining 2,301 men aged 30-79 years, and Gannagé-Yared,^13^ in a cross-sectional study on young men, reported an inverse association between the levels of SHBG and CRP, thus corroborating the inverse association between SHBG and insulin resistance. 

The current study was not intended to evaluate potential causal relationships between SHBG and metabolic syndrome. In fact, the existing data in the literature are still insufficient to confirm whether SHBG is an early marker or whether it is a component of metabolic syndrome.

In this study, a significant positive association between SHBG and TT was observed (P < 0.001), but no significant associations were observed between SHBG and BT, FT or E_2 _(Table 1). In plasma, it is known that only approximately 2% of all testosterone circulates in the free form (i.e. the fraction known as FT).^21^ In contrast, 44% is bound to SHBG, and 54% binds to albumin.^21^ Both the FT and the albumin-bound fractions are readily available to tissues. The sum of these two fractions is referred to as the BT level. Therefore, we hypothesized that a significant positive association would be observed between TT and SHBG levels.

Epidemiological studies have shown that decreased TT levels are associated with an increased risk of developing metabolic syndrome.^22^ ^,^ ^23^ These studies have suggested that low testosterone levels could contribute towards the physiopathology of metabolic syndrome and that androgen replacement therapy should be used for males with metabolic syndrome and testosterone deficiency.^24^ ^,^ ^25^ However, in a Brazilian study conducted by Callou de Sá et al.,^5^ no significant differences in the prevalence of components of metabolic syndrome (as defined according to the NCEP-ATPIII criteria)^6^ between TT terciles were observed. Similarly, in the study conducted by Bhasin et al.^14^ examining the second and third generations of the Framingham Heart Study, no significant associations between the prevalence of metabolic syndrome and TT or FT were observed in the longitudinal evaluations. Thus, these authors concluded that their data did not corroborate the hypothesis that low TT levels were independently associated with the prevalence of metabolic syndrome. 

Evaluation of SHBG as a marker for metabolic syndrome among males is important because this syndrome consists of a number of factors that confer increased risk of cardiovascular diseases, which are the main group of diseases causing death in Brazil.

## CONCLUSION

We conclude that low serum levels of SHBG were associated with higher prevalence of metabolic syndrome in our sample of adult Brazilian males aged 40-70 years. Our data should not be extrapolated to females or to other ethnic groups or age groups. Additional prospective studies to assess this association directly are required, especially with regard to the potential causal relationship between SHBG and metabolic syndrome.
